# SHED-derived exosomes promote LPS-induced wound healing with less itching by stimulating macrophage autophagy

**DOI:** 10.1186/s12951-022-01446-1

**Published:** 2022-05-21

**Authors:** Yunyi Xie, Le Yu, Zhilan Cheng, Yingying Peng, Zeyuan Cao, Beichen Chen, Yihong Duan, Yan Wang

**Affiliations:** Hospital of Stomatology, Guanghua School of Stomatology, Sun Yat-Sen University, Guangdong Provincial Key Laboratory of Stomatology, 56 Lingyuanxi Road, Guangzhou, 510055 People’s Republic of China

**Keywords:** Human exfoliated deciduous teeth (SHED), Exosomes, Macrophages, Wound healing, Itching

## Abstract

High-quality cutaneous wound healing is associated with rapid wound closure and a comfortable healing process. Currently, exosomes derived from mesenchymal stem cells displayed a prominent therapeutic effect on skin wound closure. But the therapeutic approaches for wound itching are very limited in clinical. Stem cells from human exfoliated deciduous teeth (SHED) may offer a unique exosome resource for cell-free therapeutics in potential clinical applications. Here, we investigated the common mechanisms underlying wound closure and unpleasant sensation of itching, focusing on the contribution of the SHED-derived exosome to immune response and wound itching during healing. The effects of SHED-derived exosomes on inflammatory wound healing were examined using lipopolysaccharide (LPS)-induced wounds in a mouse model. We found prolonged inflammation and distinct itch responses in skin wound tissue during LPS-induced wound healing. SHED-derived exosomes facilitated LPS-induced wound closure and relieved wound itching. Therefore, they are ideal for the treatment of wound healing. Macrophages in skin wound tissues are responsible for autophagy during wound healing. Macrophage autophagy also regulates cell proliferation, migration, and neuronal signal transduction in vitro. SHED-derived exosomes containing miR-1246 enhanced autophagy by regulating macrophage function through the AKT, ERK1/2, and STAT3 signaling pathways. Thus, SHED-derived exosomes promote wound healing with less itching in an LPS-induced wound model by stimulating macrophage autophagy, which has implications for the treatment of inflammatory wound healing.

**Graphical Abstract:**

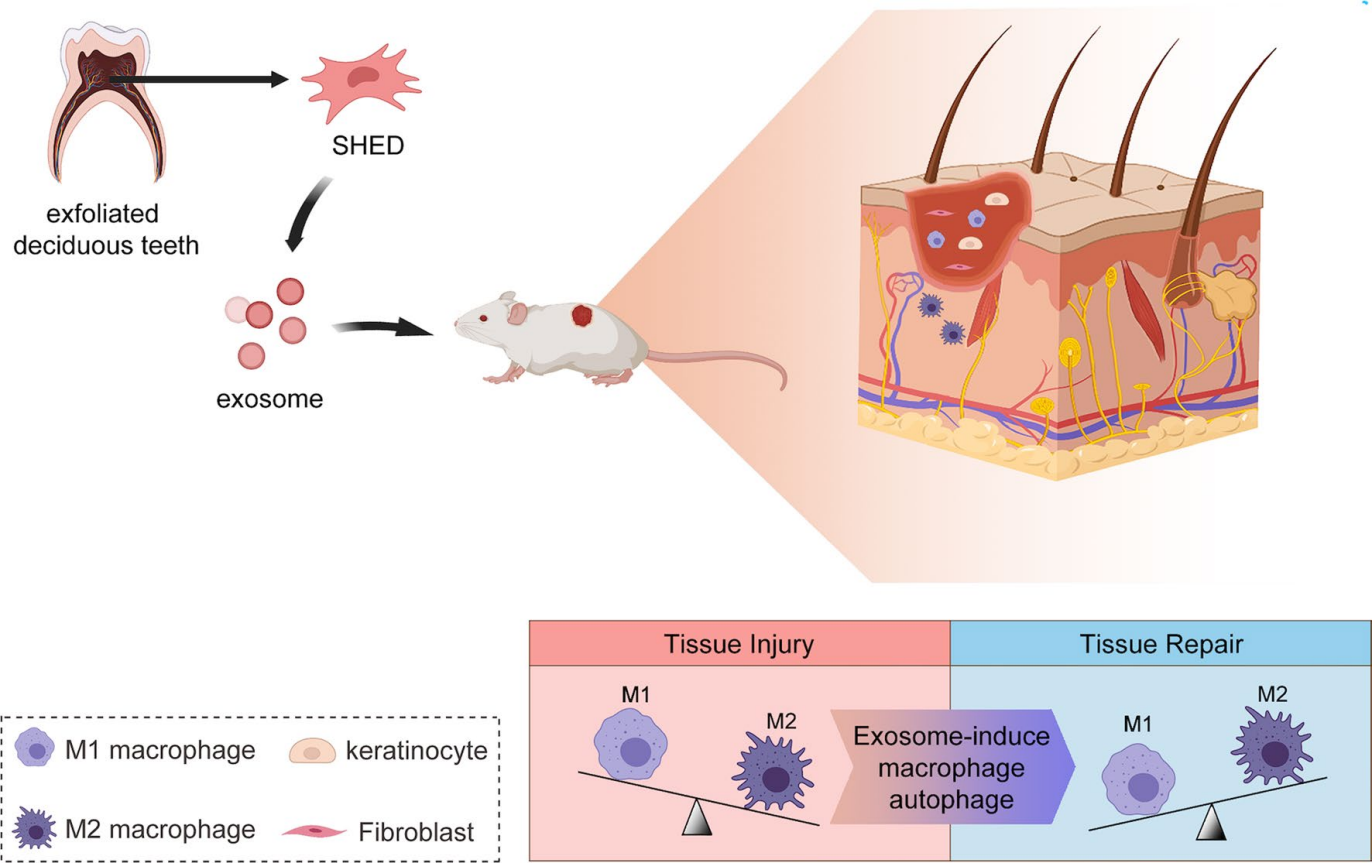

**Supplementary Information:**

The online version contains supplementary material available at 10.1186/s12951-022-01446-1.

## Background

Cutaneous wound healing is a complex process that includes acute inflammation, cell proliferation, and tissue remodeling. These stages, involving several growth factors and cytokines, are responsible for wound closure and protection against external damage [1]. Inflammatory cutaneous wounds caused by burns, trauma, diabetes, and other factors remain a major challenge for clinicians and a social burden worldwide [1, 2]. Itching may occur within a few days after wounding, which is a common problem that distresses patients, which may cause sleep disorders and affect psychosocial well-being [3, 4]. For most inflammatory wounds, itching is a common and serious complication that occurs during tissue rehabilitation and healing [3, 4]. Therefore, high-quality wound healing should not only promote wound closure, but also relieve pruritus to make patients comfortable. Conventional wound care methods, with the risk of delayed healing and pruritus, include the use of moisture-retentive dressings and adjunctive topical therapies [5]. However, the high costs and limited efficacy of these treatments have prevented their widespread clinical adoption.

When the wound healing response becomes chronic, the inflammatory response is dysregulated and tissue repair is delayed. However, multiple cell types are involved in wound healing. Macrophages, with their highly dynamic plasticity, are responsible for regulating the stages of inflammation and remodeling [6, 7]. Following tissue injury, the recruited macrophage populations reprogram and undergo marked phenotypic and functional changes in response to the release of cytokines. Different macrophage activation states have been demonstrated to play specialized and critical roles in the different phases of wound healing [8–11]. Recent studies have identified the interaction between the nervous and immune system, suggesting that cytokines released by inflammatory cells might influence itching of the skin [4, 12]. Consequently, as potentially crucial targets, macrophages release multiple inflammatory cytokines and control tissue repair responses, suggesting they could play a role in the development of wound healing and itching.

Recently, mesenchymal stem cells (MSCs) have been frequently used in the field of wound healing because of their self-renewal ability, multiple differentiation potential, and microenvironmental regulation [13, 14]. Although MSCs accelerate the resolution of wound inflammation and encourage tissue regeneration, it has been suggested that the regenerative functionality is mainly because of their paracrine actions, in which exosomes play an essential role [15–18]. Exosomes are 40–150 nm sized small membrane particles of low immunogenicity that are enriched in selected proteins, lipids, and nucleic acids. Exosomes play crucial roles in intercellular communication by transmitting biological information such as miRNAs, mRNAs, lipids and proteins to recipient cells [19]. Interestingly, several studies have demonstrated that exosomes derived from MSCs have similar immunomodulatory and regenerative functions and superior safety profiles compared to MSCs themselves [20–23]. Stem cells from human exfoliated deciduous teeth (SHED) possess excellent pluripotency and self-renewal capacity. Moreover, SHED are abundant and easily accessible source of MSCs. SHED are known to differentiate into neural cells and are capable of high immunoregulatory activity [24, 25]. However, the effect and underlying mechanisms of SHED-derived exosomes (SHED-Exo) on wound healing and itching remain unclear.

Here, we utilized an experimental mouse model of wound healing to investigate the effects of SHED-Exo on lipopolysaccharide (LPS)-induced wound healing and itching. We confirmed the healing-promoting and itch-suppressing effects of SHED-Exo on the skin and elucidated that macrophage autophagy regulated tissue regeneration and neuron sensitivity. Importantly, we also showed that exosomal miR-1246 stimulated macrophage autophagy via the AKT, ERK1/2, and STAT3 signaling pathways.

## Results

### Characterization of SHED

The identification of SHED was confirmed by a colony formation assay, surface markers by flow-cytometric analysis, tissue origin by immunofluorescence, and multiple differentiation by staining assays (Additional file 1: Fig. S1). SHED showed a spindle-like morphology and clearly formed colonies on day 14 after seeding at a low density (Additional file 1: Fig. S1a). Flow cytometry showed that SHED expressed CD44 (99.5%), CD90 (99.4%), and CD105 (99.49%), but negatively expressed CD34 (0.56%), CD45 (0.68%), and HLA-DR (0.15%) (Additional file 1: Fig. S1b). The immunofluorescence results showed that SHED expressed the mesenchymal marker vimentin but barely expressed the epithelial marker cytokeratin 18 (Additional file 1: Fig. S1c). Multiple differentiation, including osteogenesis and adipogenesis, was performed to assess pluripotency. After incubation in osteogenic differentiation medium, SHED demonstrated osteocyte features stained with alizarin red (Additional file 1: Fig. S1d). Adipogenesis was evaluated by Oil Red O staining after 3 weeks of incubation in adipogenic differentiation medium (Additional file 1: Fig. S1d). Taken together, these results confirmed that SHED possessed MSC properties and pluripotency.

### Characterization of SHED-Exo

TEM, western blotting, nanoparticle tracking analysis (NTA), zeta potential analysis and nano-flow cytometry were performed to identify the exosomes isolated from the supernatant of SHED. TEM verified that SHED-Exo exhibited a cup-shaped morphology with a size less than 150 nm (Additional file 1: Fig. S2a). NTA analysis revealed that the size of the SHED-Exo was approximately 40–150 nm with a mean diameter of 92.0 nm (Additional file 1: Fig. S2c), which was consistent with previously reported size distributions [19]. Western blotting indicated that CD63, CD81, CD9, TSG101, and ALIX were highly enriched in SHED-Exo and these exosomes were negative for α-tubulin and albumin (Additional file 1: Fig. S2b) [26]. To examine the charge density distribution around the exosome, which is a parameter known as the zeta potential, SHED-derived exosomes were measured using a zeta potential analyzer. Results showed that SHED-derived exosomes demonstrated zeta potential was − 29.57 ± 0.74 mV, which is consistent with the zeta potential range of exosomes reported previously (Additional file 1: Fig. S2d) [27]. Nano-flow cytometry results showed that the classical exosomes protein markers such as CD63, CD81, and CD9 were expressed in SHED-Exo (Additional file 1: Fig. S2e), which agreed well with previously reported values [28]. These data revealed that the isolated SHED-Exo possessed characteristics identical to those of exosomes [26].

### Application of LPS-induced delayed wound healing with itching responses

**Fig. 1:**
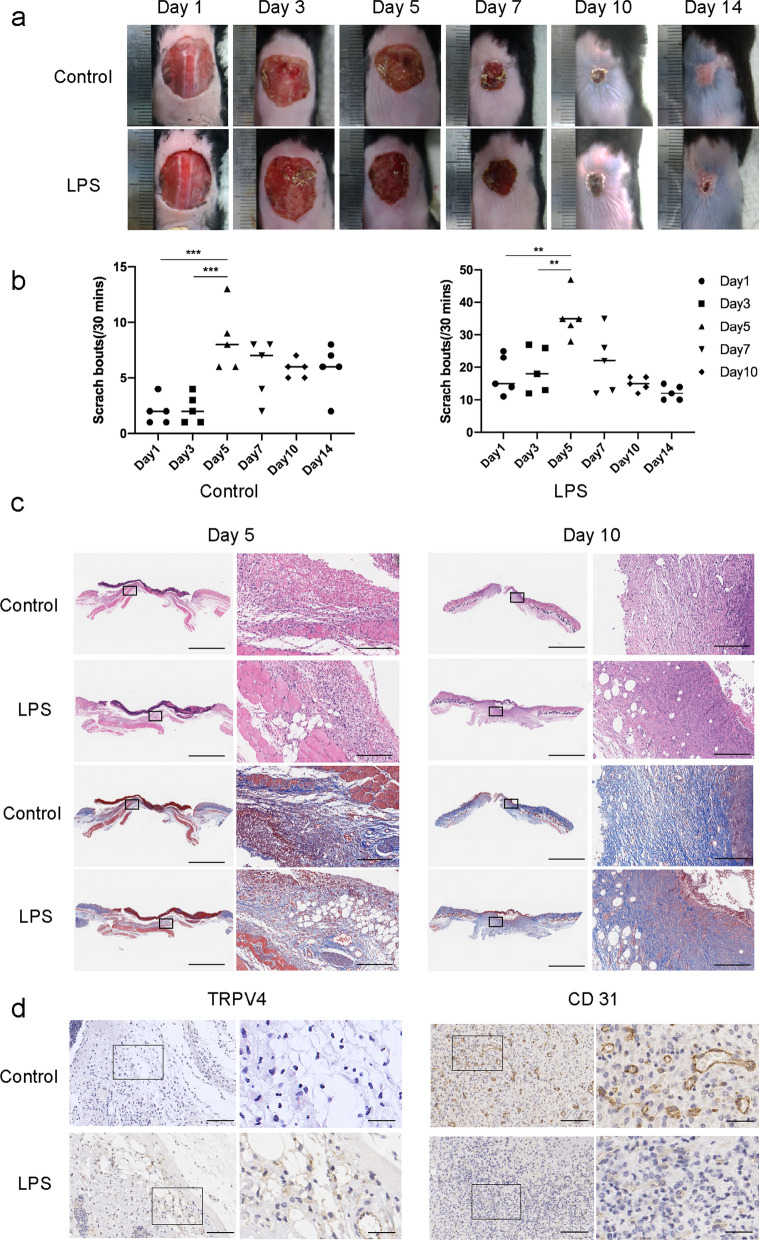
Application of LPS-induced delayed wound healing with itching. **a** Representative photographs showing macroscopic excisional wound closure of C57BL6 mice subcutaneously injected with PBS (Control), or 0.5 mg/kg LPS (LPS). **b** Itching behaviors of mice with cutaneous wounds during wound healing. n = 5. *P < 0.05; **P < 0.01; ***P < 0.001. **c** Representative H&E and Masson-stained sections of PBS- or LPS-injected incisional wounds. Scale bars (low power field), 3 mm. Scale bars (high power field), 200 µm. **d** Representative immunohistochemistry of TRPV4 on day 5 and CD31 on day 10 within PBS- or LPS-injected incisional wounds. Scale bars (low power field), 100 µm. Scale bars (high power field), 30 µm

To determine the effect of prolonged inflammation on cutaneous wound healing, we monitored wound closure and pruritus elicited by an LPS-induced skin wound model in mice. A previous study reported that wound administration of LPS as a model of infection in burn injury led to impaired cutaneous healing [29, 30]. For wound closure, we evaluated the macroscopic appearance of the skin wounds at 0–14 d post-wounding. We observed that the LPS-treated wounds had delayed healing compared to the control wounds (Fig. 1a). On day 14, the wounds in control mice almost completely healed, whereas those treated with LPS did not heal. Next, we evaluated pruritus in mice using scratch bouts and found that scratching responses of LPS-treated mice were much more serious than those of control wound mice and no-wound mice (Fig. 1b). Moreover, the scratch bouts were maximal on day 5 post-wounding. During wound healing, histopathological changes were observed in the different skin samples. To further examine the tissue formation and remodeling of the wound, H&E, Masson, and immunohistochemical staining were performed. H&E staining showed that wounds in the control group displayed faster closure and fewer inflammatory cells than the LPS-treated groups (Fig. 1c). Masson staining revealed that LPS-treated wounds exhibited impaired recovery, with immature fibrous tissues and disorganized fibroblasts. TRPV4 is a non-selective calcium-permeable cation channel that can be used to confirm the degree of neuron sensitivity and skin itching [31, 32]. The results showed that LPS-treated wounds were vastly different from control wounds, with significantly increased TRPV4 appearance at day 5 post-wounding (Fig. 1d). CD31 is expressed at the borders between endothelial cells and can be used to assess tissue vascularization [33]. Moreover, significantly loosely packed collagen fibers and less CD31 were present in LPS day 10 wounds compared to control mice. These data suggest that LPS treatment leads to a prolonged inflammatory response and impaired wound healing with distinct itching.

### SHED-Exo promote LPS-induced delayed wound healing

**Fig. 2:**
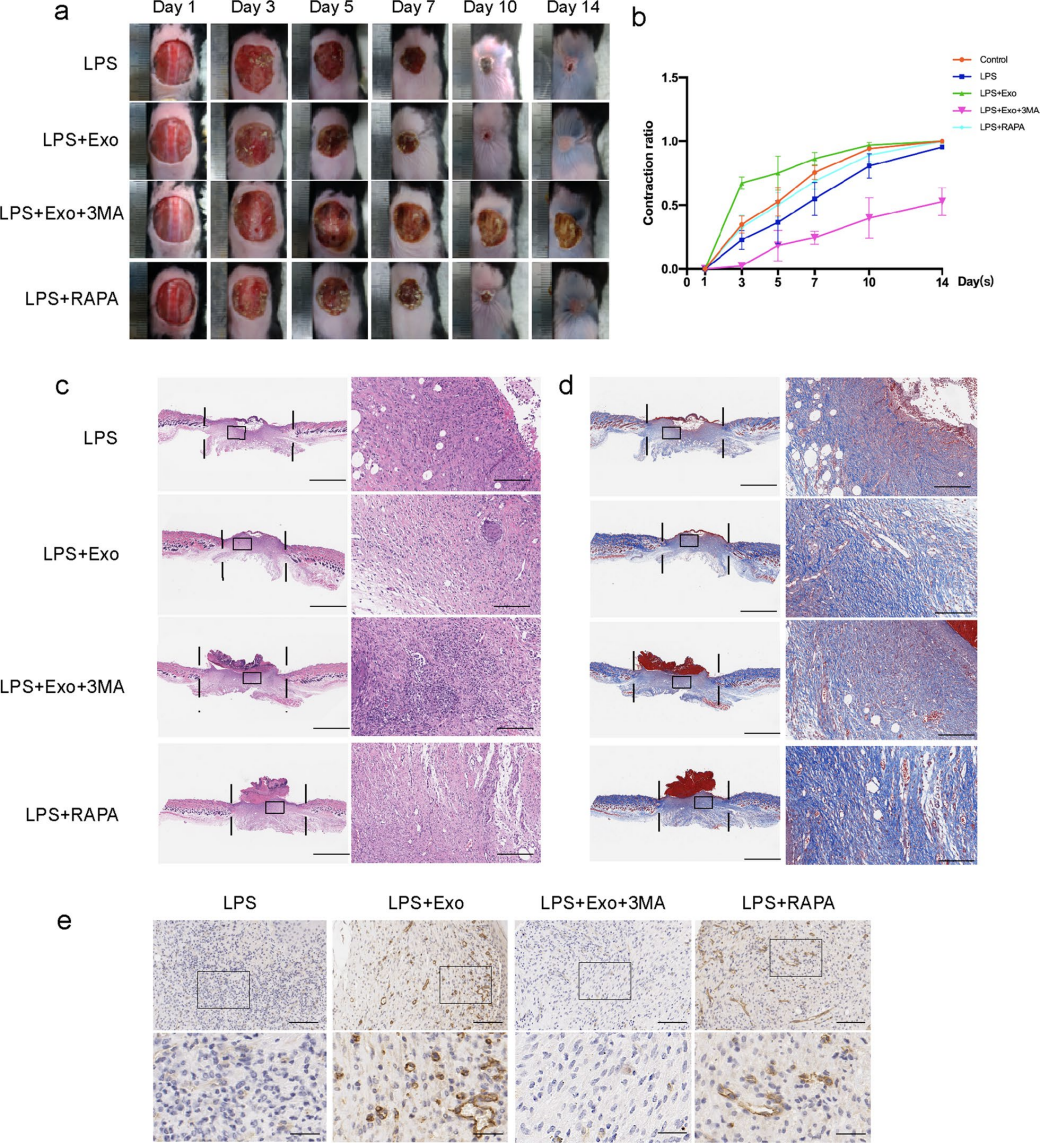
SHED-Exo promote LPS-induced chronic wound healing. **a** Representative wound images from mice treated with LPS, LPS containing exosomes (LPS + Exo), LPS containing exosomes and 3-MA(LPS + Exo + 3MA), and LPS containing rapamycin (LPS + RAPA) over 14 days of healing period. **b** Comparison of the percentages of the open wound size over 10 days healing period between LPS, LPS + Exo, LPS + Exo + 3MA and LPS + RAPA groups. n = 5. **c** H&E staining images of full-thickness wounds on days 10, arrows indicate newly formed dermal appendages, scale bar: 1000 μm. **d** Masson staining images of full-thickness wounds on days 10, arrows indicate newly formed dermal appendages. Scale bars (low power field), 3 mm. Scale bars (high power field), 200 µm. **e** Representative immunohistochemistry of CD31 within incisional wounds. Scale bars (low power field), 100 µm. Scale bars (high power field), 30 µm

To confirm the effect of SHED-Exo on LPS-induced wound healing, we locally injected LPS and exosomes into the cutaneous wounds of LPS-treated mice. The results revealed faster-wound closure in the SHED-Exo group than in the LPS group on days 5, 7, 10, and 14 (P < 0.05) (n = 5) (Fig. 2a, b). H&E and Masson staining were used to assess the healing pathology of cutaneous wounds. The results showed that SHED-Exo-treated wounds showed markedly dampened local recruitment of inflammatory cells and less of a necrotic appearance on days 5 and 10 after wounding (Fig. 2c). Masson staining results showed that the SHED-Exo group displayed more structurally developed collagen fibers than the LPS group on day 10 (Fig. 2d). These results suggested that SHED-Exo can partially ameliorate the delayed wound healing process in an LPS-induced mouse model.

Previous studies have reported that autophagy, which coordinates multicellular immunity and limited inflammatory pathologies, plays a fundamental role in immunity [34]. To confirm that autophagy is responsible for the prolonged inflammatory response during wound healing, we predicted that the administration of additional 3-MA or rapamycin would further affect these responses. Thus, we injected 3-MA and rapamycin into wounds. In agreement with our hypothesis, the wound healing rate in the SHED-Exo and rapamycin treatment groups was significantly higher than that in the LPS group, whereas 3-MA reversed the effect of SHED-Exo (P < 0.05) (n = 5) (Fig. 2b). Additionally, wounds treated with SHED-Exo and rapamycin showed abundant and relatively well-arranged collagen fibers on day 10 (Fig. 2d). However, 3-MA inhibited the effects of SHED-Exo, characterized by reduced collagen deposition at the wound site. Unlike the LPS and 3-MA groups, which exhibited less angiogenesis, abundant granulation tissue formed with thinner and more layers in the wound gap of the SHED-Exo and rapamycin groups (Fig. 2d). Moreover, SHED-Exo-treated wounds exhibited the most abundant granulation tissue and the shortest wound length. Collectively, these results suggested that SHED-Exo facilitated wound closure and promoted tissue regeneration, and these positive effects may be related to autophagy.

### SHED-Exo relieve itch responses during wound healing

**Fig. 3:**
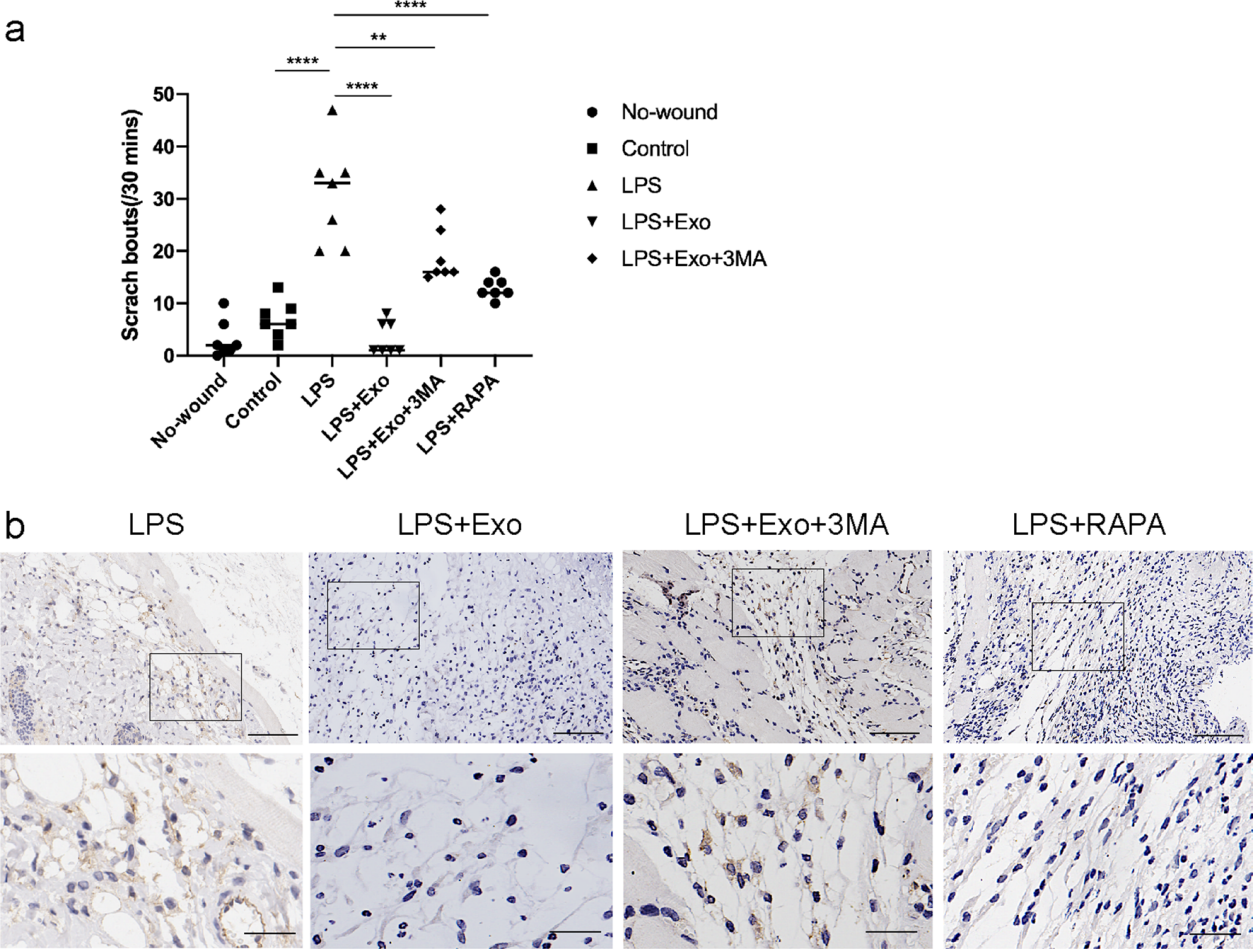
SHED-Exo relieve itch responses during wound healing. **a** Scratching behaviors were observed in mice before and on the 5th day of wound healing. Data are from two independent experiments. n = 7. *P < 0.05; **P < 0.01; ***P < 0.001. **b** Representative immunohistochemistry of TRPV4 within incisional wounds on day 5. Scale bars (low power field), 100 µm. Scale bars (high power field), 30 µm

To further investigate whether SHED-Exo regulate itch responses during wound healing, which could be associated with the autophagy level, we observed scratching responses and TRPV4 appearance on day 5 of wound healing. LPS-treated mice displayed a higher number of scratching bouts compared to the control and no-wound animals (Fig. 3a). Additionally, mice treated with SHED-Exo scratched much less than the LPS-treated mice on the 5th day of healing. Consistent with this result, SHED-Exo inhibited LPS-induced upregulation of TRPV4 expression in the skin, whereas 3-MA reversed the effects of SHED-Exo, indicating that these exosome-mediated effects are involved with autophagy (Fig. 3b). Collectively, these results suggested that SHED-Exo relieve itching associated with autophagy during wound healing.

### Macrophage-specific autophagy is enhanced by SHED-Exo

To investigated the main cell type to take up SHED-Exo at wound sites, we measured the exosomes uptake efficiency of different cell types at the wound site. The unwounded mice and wounded mice were subcutaneously injected with fluorescence dye‐labeled SHED-Exo. Wound tissue sections on 48 h post-operation were stained with F4/80 as a marker for macrophages. Results showed that most of the PKH-67 labeled exosomes in the wound tissues were taken up by F4/80 positive cells, indicating that SHED-Exo were mainly absorbed by macrophages in the wound healing process (Additional file 1: Fig. S4a). Furthermore, the confocal microscopy images and fluorescence intensity results showed a significant increase in cellular attachment and internalization of SHED‐Exo in THP-1 macrophages within 4 h, which is consistent with in vivo results (Additional file 1: Fig. S4b, c). We confirmed that SHED‐Exo was preferentially taken up by macrophages at the wound site. Notably, SHED‐Exo exhibited the highest cellular uptake efficiency in the macrophages, indicating that SHED‐Exo is recognizable to macrophages by their phagocytosis function.

**Fig. 4:**
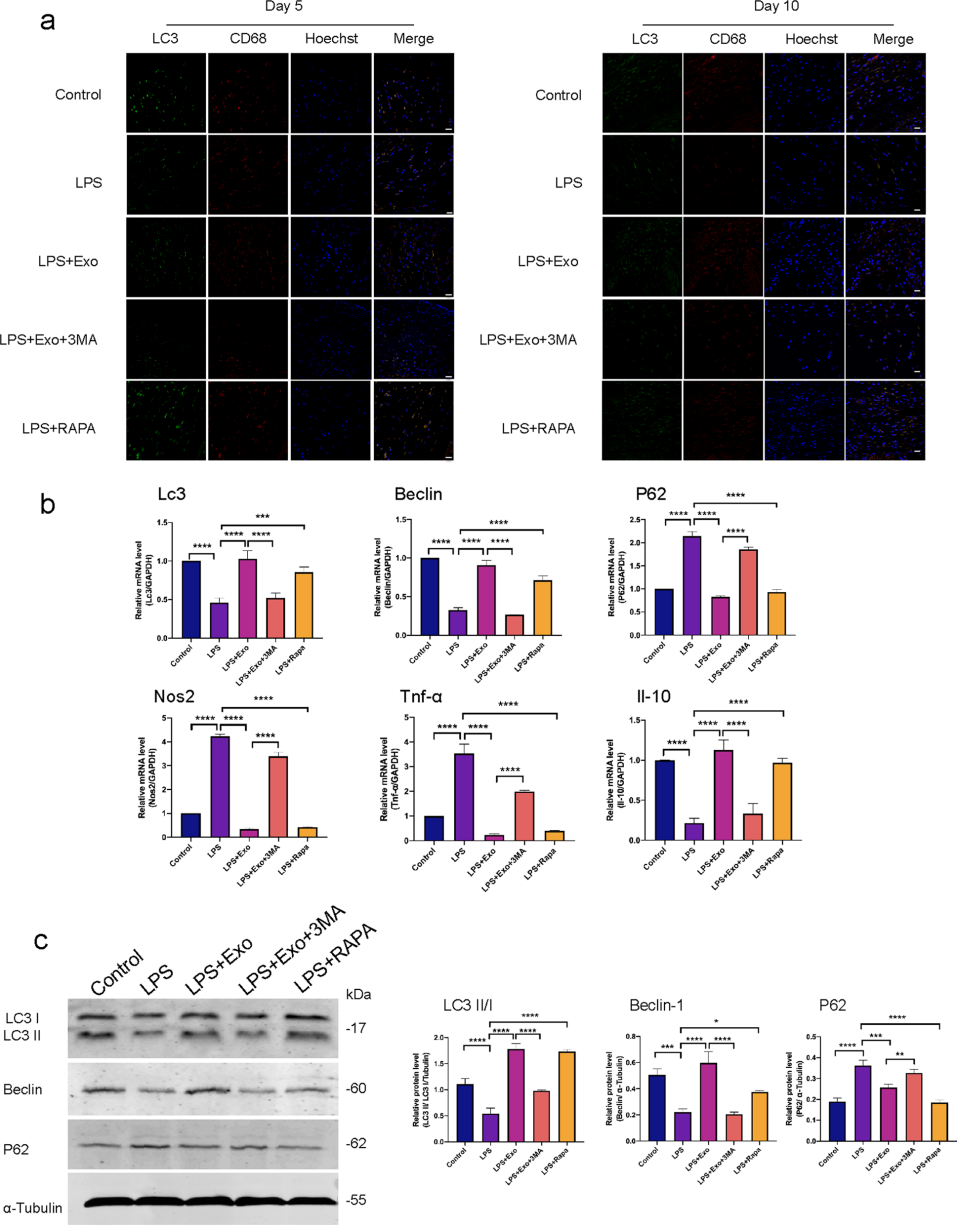
Macrophage-specific autophagy is enhanced by SHED-Exo. **a** Representative images of skin tissue sections stained for immunofluorescent detection of LC3 (green), CD68 (red), (yellow is indication of co-localization), and hoechst (blue). Scale bars, 50 µm. **b** Tissue gene expression levels of autophagy (*Lc3*, *Beclin1*, and *p62*) and stemness (*Nos2*, *Il10*, and *Tnf-α*) markers per group. n = 3. *P < 0.05; **P < 0.01; ***P < 0.001. **c** Western blot results and relative expression levels of LC3, Beclin1, and P62 proteins per treatment group. n = 3. *P < 0.05; **P < 0.01; ***P < 0.001

To clarify the mechanism by which cell autophagy regulates wound healing, we examined the cell types of autophagy in skin wounds. Wound tissue sections on days 5 and 10 post-operation were stained with LC3 as a marker for autophagy and CD68 as a marker for macrophages. SHED-Exo and rapamycin treatment significantly increased the number of LC3 positive cells, whereas 3-MA treatment reversed the effects of exosomes (Fig. 4a). In addition, most of the LC3 positive cells in the wound tissues were CD68 positive cells, indicating that macrophages were the main cell type in the wound healing process.

To analyze whether the autophagy level changed in wounds, RT-qPCR was performed to evaluate autophagy-related gene expression in the skin tissue. The SHED-Exo-treated group had higher *LC3* and *Beclin1* expression levels and lower *p62* expression levels than the LPS-treated group, whereas 3-MA treatment reversed the effects of exosomes (Fig. 4b). The western blot results were consistent with the RT-qPCR results. Compared with the control group, the expression of autophagy-related proteins LC3 and Beclin1 decreased and the expression level of P62 increased in the skin tissues of LPS-treated mice. SHED-Exo and rapamycin inhibited the effect of LPS and increased the protein expression levels of LC3 and Beclin1, whereas the protein expression level of P62 decreased (Fig. 4c). Taken together, these results demonstrated that treatment with SHED-Exo mediated anti-inflammatory effects in the skin wound via modulation of autophagy in macrophages.

### Macrophages are responsible for wound re-epithelialization and sensory neuron sensitivity

**Fig. 5:**
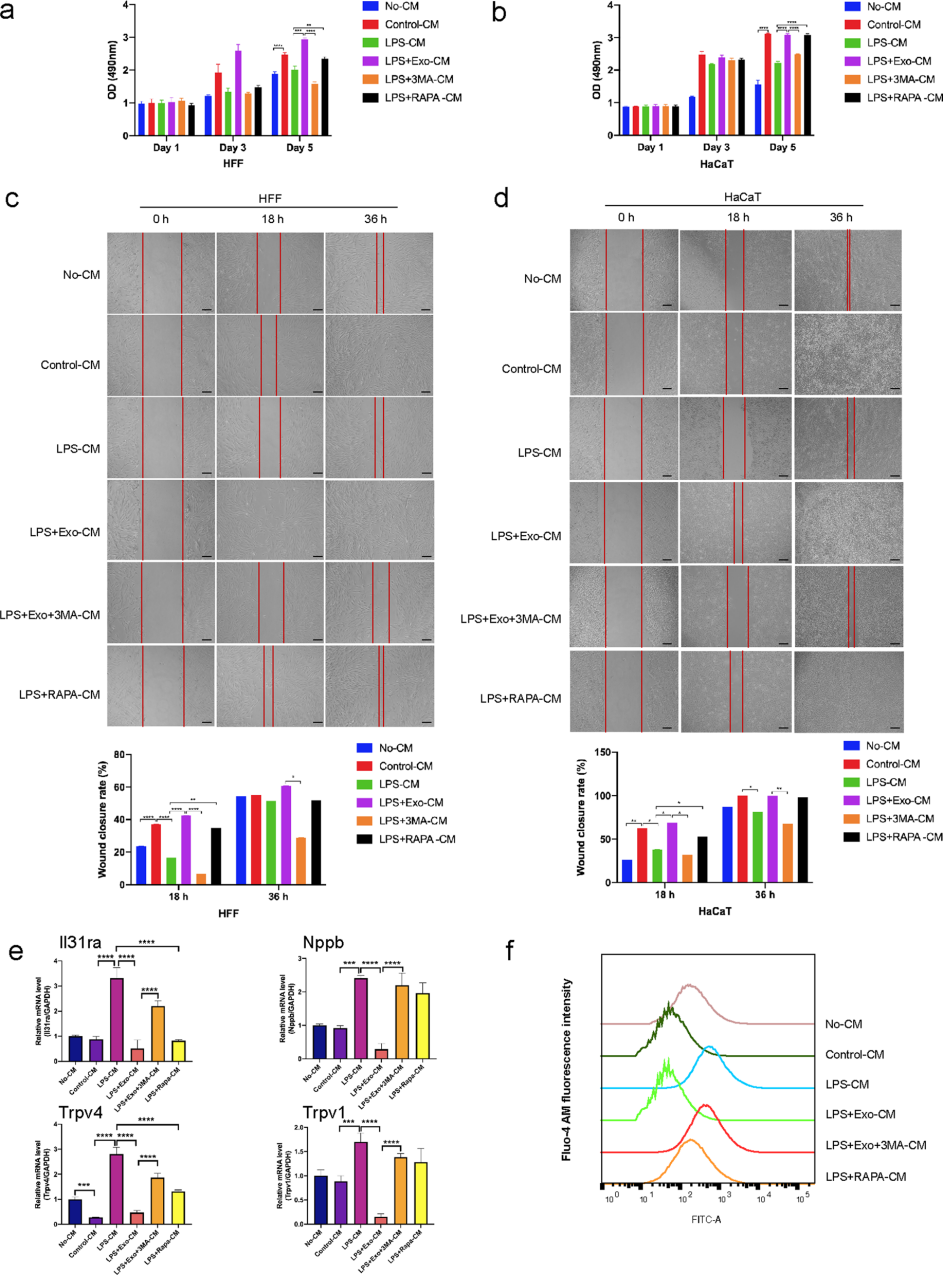
Macrophages are responsible for itch responses and wound healing. **a** HFF cells were incubated with DMEM (No-CM), conditioned medium from THP-1 macrophages (Control-CM), THP-1 macrophages treated with LPS (LPS-CM), THP-1 macrophages treated with LPS and exosomes (LPS + Exo-CM), THP-1 macrophages treated with LPS, exosomes and 3-MA (LPS + Exo + 3MA-CM) and THP-1 macrophages treated with LPS and rapamycin (LPS + RAPA-CM). MTS assay and corresponding proliferative cell rate (%) of HFF cells. n = 3. *P < 0.05; **P < 0.01; ***P < 0.001. **b** HaCaT cells were incubated with DMEM (No-CM), conditioned medium from THP-1 macrophages (Control-CM), THP-1 macrophages treated with LPS (LPS-CM), THP-1 macrophages treated with LPS and exosomes (LPS + Exo-CM), THP-1 macrophages treated with LPS, exosomes and 3-MA (LPS + Exo + 3MA-CM) and THP-1 macrophages treated with LPS and rapamycin (LPS + RAPA-CM). MTS assay and corresponding proliferative cell rate (%) of HaCaT cells. n = 3. *P < 0.05; **P < 0.01; ***P < 0.001. **c** Wound closure rate (%) of HFF cells per treatment group. Scale bars, 100 µm. **d** Wound closure rate (%) of HaCaT cells per treatment group. Scale bars, 100 µm. **e** SH-SY5Y cells were incubated with DMEM/F12 medium (No-CM), conditioned medium from THP-1 macrophages (Control-CM), THP-1 macrophages treated with LPS (LPS-CM), THP-1 macrophages treated with LPS and exosomes (LPS + Exo-CM), THP-1 macrophages treated with LPS, exosomes and 3-MA (LPS + Exo + 3MA-CM) and THP-1 macrophages treated with LPS and rapamycin (LPS + RAPA-CM). Relative mRNA expression levels of neuron sensitivity markers, *Il31ra*, *Nppb*, *Trpv4*, and *Trpv1*, in SH-SY5Y per treatment group. n = 3. *P < 0.05; **P < 0.01; ***P < 0.001. **f** The calcium transient in capsaicin (500 nM) was observed in SH-SY5Y per treatment group

To gain further insight into macrophage-mediated wound healing, we postulated that SHED-Exo could be internalized into macrophages to regulate epithelial cells and neurons adjacent to the wound. Moreover, 3-MA reversed the effects of SHED-Exo in a mouse model, which led us to hypothesize that exosome-mediated macrophage autophagy might account for the orchestrated migration and proliferation of epithelial cells and neuron sensitivity. Thus, we employed the human THP-1 cell line as a normal mononuclear macrophage line to further explore whether macrophages could regulate wound healing and itching in vitro. THP-1 cells were incubated with PMA to induce differentiation into M0 macrophages, which were appropriately characterized by adherent morphology and the elevated expression of the macrophage marker CD68 (Additional file 1: Fig. S3a). When co-cultured with macrophages, SHED-Exo labeled with PKH67 (green) were internalized by unstained macrophages (Additional file 1: Fig. S3b). To confirm the key role of macrophages on epithelial cells and neurons, the supernatants of THP-1 macrophages with different treatments were collected for culture with HaCaT, HFF, and SY-SY5Y cells. The results showed that HaCaT and HFF cells cultured with supernatants from THP-1 macrophages proliferated and migrated much faster than those cultured with DMEM (Fig. 5a–d). The proliferation and migration ability of the HaCaT and HFF cells in the LPS group was significantly compromised, whereas supernatants from the SHED-Exo group enhanced cell proliferation and migration. Together, these data suggested that macrophages facilitated re-epithelialization by promoting the proliferation and migration of keratinocytes and fibroblasts.

Next, we explored the expression of signal transduction-related genes in SH-SY5Y cells cultured with supernatants from THP-1 macrophages. We found that expression of *Il31ra*, *Nppb*, *Trpv4*, and *Trpv1* was upregulated in SH-SY5Y cells of the LPS group, whereas the expression of these genes in cells of the SHED-Exo group did not change significantly (Fig. 5e). These results indicated that macrophages are responsible for sensory neuron sensitivity. To further test this hypothesis, we observed calcium influx in SH-SY5Y cells cultured with THP-1 macrophage supernatants. The results showed that treatment of supernatants from LPS-stimulated macrophages potentiated capsaicin (500 nM)-stimulated calcium influx in SH-SY5Y cells (Fig. 5f). However, the calcium influx in the SHED-Exo group did not increase significantly. These results suggested that macrophages could increase gene expression of key signal transduction molecules in sensory neurons and sensitize these nerves.

### SHED-Exo enhanced autophagy regulates macrophage function

**Fig. 6:**
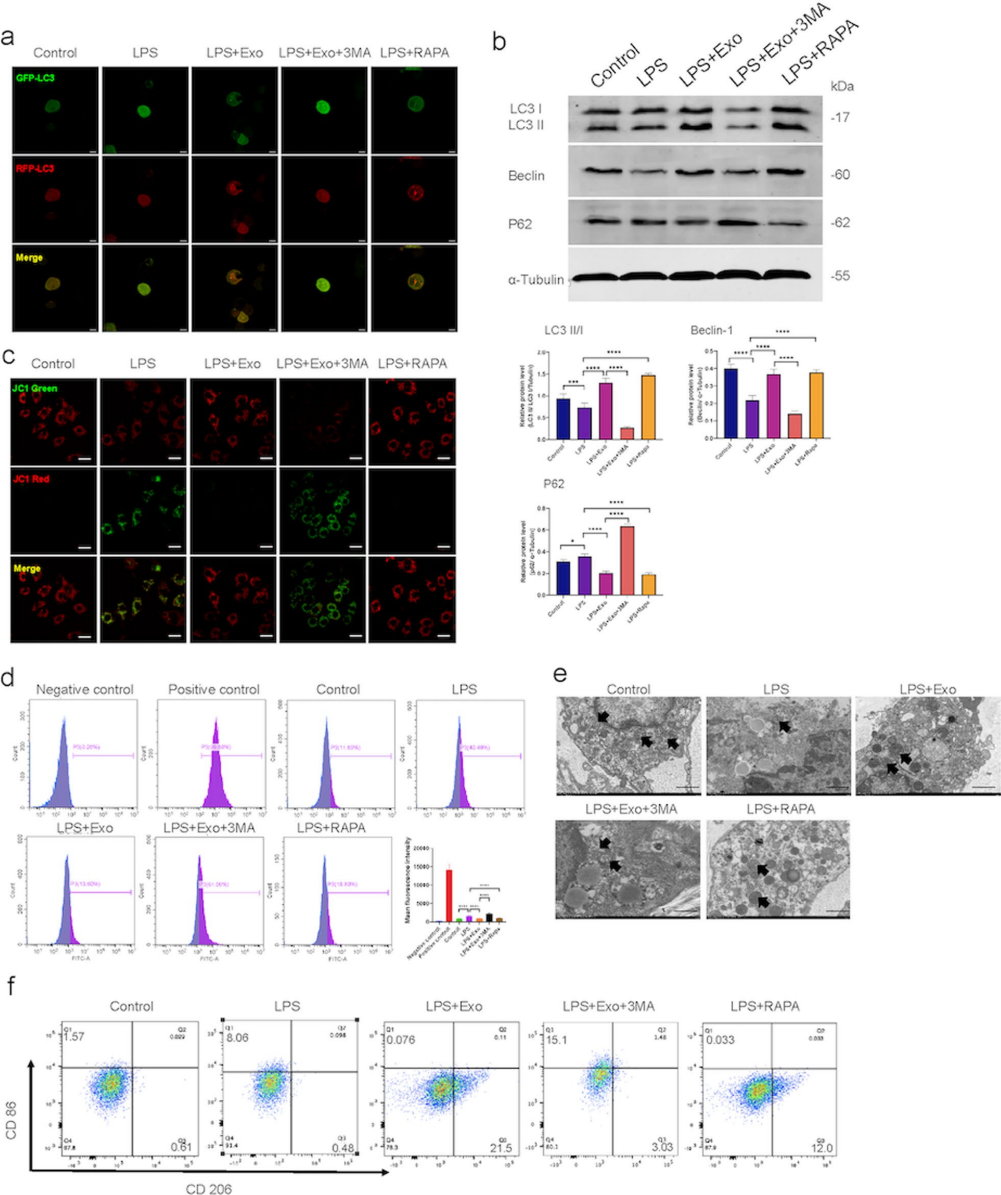
SHED-Exo enhanced autophagy regulates macrophage function. **a** THP-1 cells treated with 1 μg/mL LPS (LPS), THP-1 treated with LPS and 20 ng/mL exosomes (LPS + Exo), THP-1 treated with LPS, exosomes and 5 mM 3-MA (LPS + Exo + 3MA) and THP-1 treated with LPS and 100 μM rapamycin (LPS + RAPA) for 12 h after PMA stimulation. Representative images of autophagy flux levels in THP-1 cells per treatment group transfected with mRFP‐GFP‐LC3. Scale bars, 10 µm. **b** Western blot results and relative expression levels of LC3, Beclin1, and P62 proteins per treatment group. n = 3. *P < 0.05; **P < 0.01; ***P < 0.001. **c** Representative images of mitochondrial membrane potential in THP-1 cells per treatment group as detected by a JC-1 probe. The J-aggregates produced red fluorescence (JC-1 red); the monomer produced green fluorescence (JC-1 green). Scale bars, 20 µm. **d** Flow cytometry results of ROS levels and mean fluorescent intensity in THP-1 cells per treatment group. n = 3. *P < 0.05; **P < 0.01; ***P < 0.001. **e** The morphology of mitochondria in THP-1 cells was analyzed by TEM. Scale bars, 1 µm. **f** Flow cytometry results for surface markers of macrophage polarization

To establish a link between exosome-enhanced autophagy and macrophage function, in vitro studies were performed with THP-1 derived macrophages. We first determined whether macrophage autophagy had a differential response to SHED-Exo by mRFP-GFP-LC3 analysis and western blotting. THP-1 cells were transfected with a lentiviral vector expressing mRFP‐GFP‐LC3 to study autophagosome formation. The immunofluorescence data showed that LPS treatment resulted in enhanced accumulation of green puncta in macrophages, suggesting a decrease in autophagic flux (Fig. 6a). However, SHED-Exo and rapamycin treatment groups showed an increase in red and yellow puncta, indicating an increase in either autolysosomes or autophagosomes, which enhanced the autophagic flux. In contrast, 3-MA-treated cells showed a significantly decreased mRFP‐GFP‐LC3 puncta compared with the SHED-Exo group. Consistently, western blot data revealed that LC3‐II accumulation was further increased in the presence of SHED-Exo in culture medium (Fig. 6b), confirming that SHED-Exo elicited an inhibitory effect on the LPS‐induced blockage of the autophagy flux.

To evaluate the effects of exosomes on macrophage function, mitochondrial function, and polarization states were evaluated in vitro. Inflammation can cause mitochondrial dysfunction, consequently regulating macrophage activation [35, 36]. The mitochondrial membrane potential was calculated using the JC-1 mitochondrial membrane potential assay. The results showed that the mitochondrial membrane potential decreased in LPS- and 3-MA-treated cells compared to the control group (Fig. 6c). However, SHED-Exo and rapamycin-treated THP-1 cells exhibited a higher intensity of red fluorescence and a lower intensity of green fluorescence, indicating a higher mitochondrial membrane potential. The oxidative status of THP-1 cells was assessed by measuring ROS production. The results showed that the ROS level in LPS-stimulated cells was increased. THP-1 cells in the SHED-Exo and RAPA groups had low ROS levels, whereas cells treated with 3-MA had higher intracellular ROS levels (Fig. 6d). Mitochondrial ultrastructural changes were observed using TEM (Fig. 6e). No obvious ultrastructural changes were noted in the mitochondrial cristae or matrix density in the control group. When treated with LPS, a loss of cristae integrity, deformed endoplasmic reticulum, and lipid droplet accumulation were observed. These alterations could be alleviated when THP-1 cells were treated with SHED-Exo or RAPA. However, 3-MA reversed the mitochondrial protective effects of SHED-Exo. Next, we examined the effects of SHED-Exo on macrophage polarization states. The expression of the M2 marker CD206 was significantly increased, and the M1 marker CD86 was decreased in macrophages incubated with SHED-Exo compared to that in macrophages treated with LPS or cells incubated with 3-MA (Fig. 6f). Collectively, these results suggested that SHED-Exo regulated macrophage function by manipulating autophagy.

### SHED-Exo containing miR-1246 induced autophagy in macrophages via the AKT, ERK1/2, and STAT3 signaling pathway

**Fig. 7:**
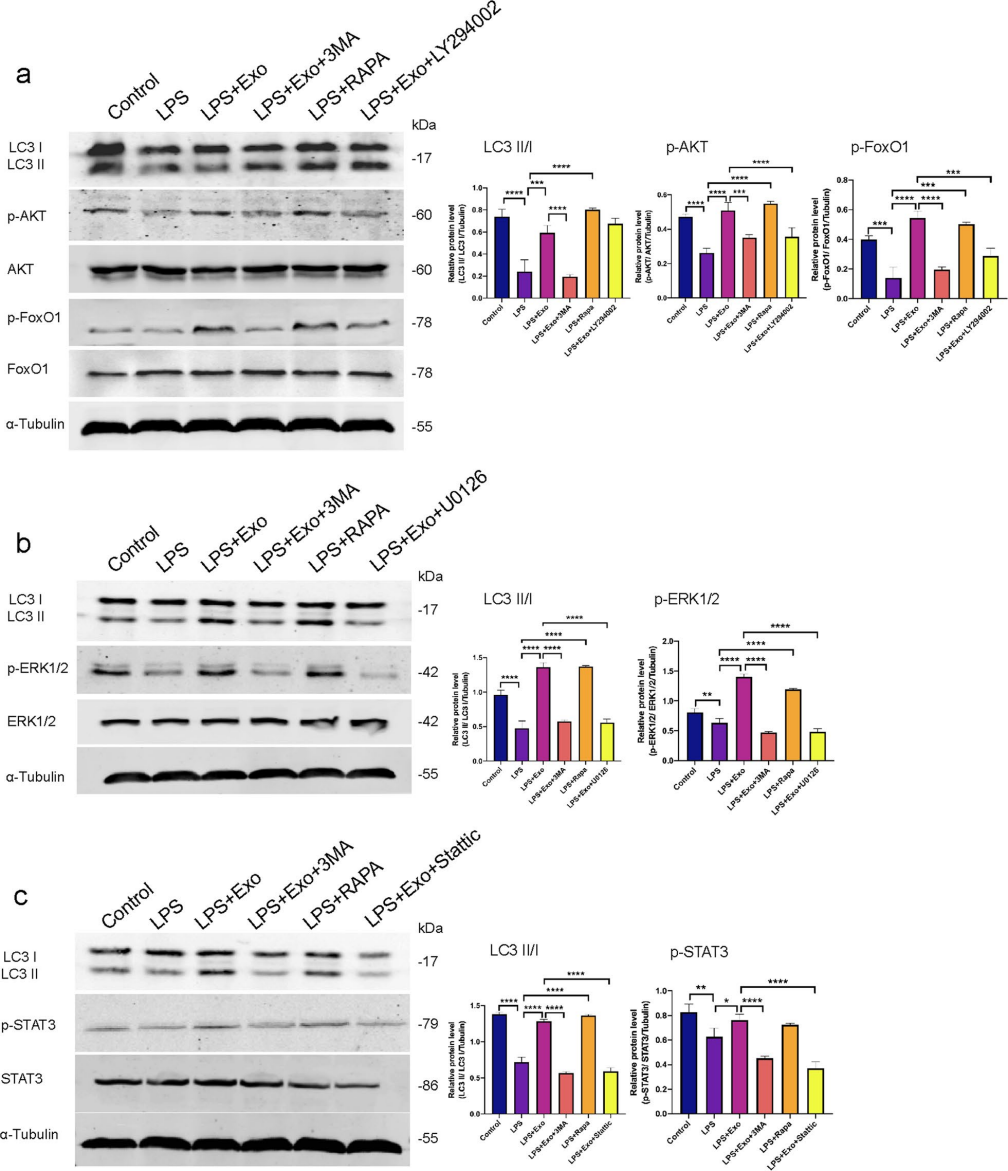
SHED-Exo induced autophagy in macrophages via the AKT, ERK1/2, and STAT3 signaling pathway. **a** THP-1 cells treated with 1 μg/mL LPS, 20 ng/mL exosomes, 5 mM 3-MA, 100 μM rapamycin, 10 μM LY294002, 10 μM U0126, and 10 μM Stattic after PMA stimulation. Western blot results and relative expression levels of LC3, AKT, and FoxO1 proteins per treatment group. n = 3. *P < 0.05; **P < 0.01; ***P < 0.001. **b** Western blot results and relative expression levels of LC3 and ERK1/2 signaling pathway-related proteins per treatment group. n = 3. *P < 0.05; **P < 0.01; ***P < 0.001. **c** Western blot results and relative expression levels of LC3 and STAT3 signaling pathway-related proteins per treatment group. n = 3. *P < 0.05; **P < 0.01; ***P < 0.001

To determine the mechanism of SHED-Exo responsible for macrophage autophagy, the AKT, ERK1/2, and STAT3 signaling pathways were assessed using western blot analysis. The results showed that LPS-treated macrophages had significantly decreased expression of LC3II/I. However, SHED-Exo and RAPA treatments resulted in increased expression of LC3II/I. Furthermore, western blot analysis showed that SHED-Exo promoted the phosphorylation of AKT, FoxO1, and ERK1/2 (Fig. 7a, b). Therefore, we speculated that SHED-Exo promoted autophagy of macrophages via the AKT and ERK1/2 pathways. In addition, the proteins related to the STAT3 pathway were activated and had significantly higher expression in the SHED-Exo-treated groups than in the LPS-treated group (Fig. 7c). AKT, ERK1/2, and STAT3 inhibitors have been used separately in in vitro experiments. THP-1 macrophages were treated with RPMI 1640 medium containing 10 μM LY294002 for 6 h to block the AKT pathway, 10 μM U0126 for 6 h to block the ERK1/2 pathway, and 5 μM Stattic for 6 h to block the STAT3 pathway [37]. The results showed that SHED-Exo promoted the phosphorylation of AKT, ERK1/2, and STAT3 compared to the LPS group. The phosphorylation levels of AKT, ERK1/2, and STAT3 decreased when cells were treated with the corresponding inhibitors, whereas the expression of LC3II/I in cells was increased by treatment with LY294002, U0126, and Stattic.

**Fig. 8:**
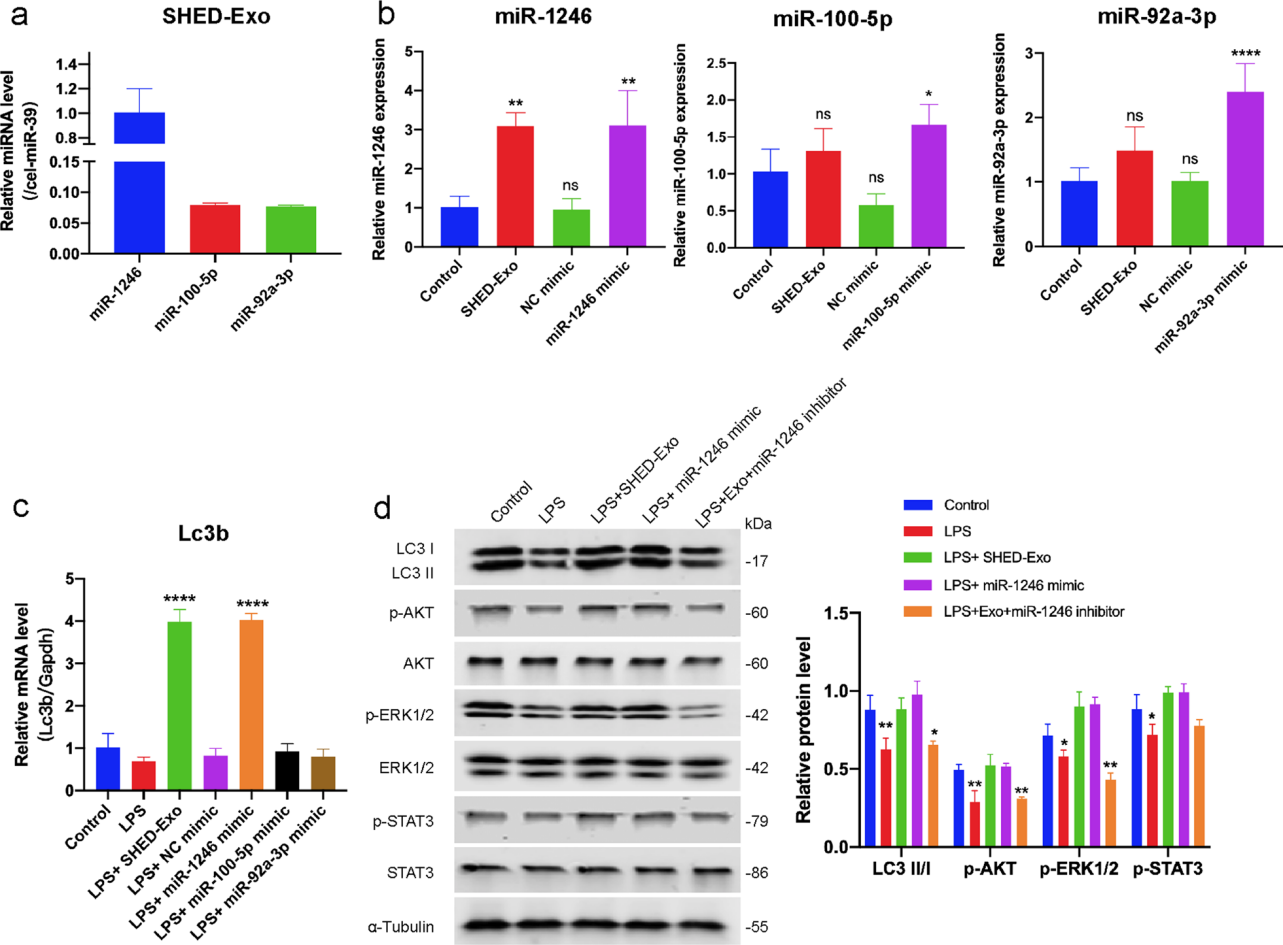
miR-1246 as a SHED-Exo-enriched miRNA that stimulating macrophage autophagy. **a** The miRNA expression levels of SHED-derived exosomes were determined by qRT-PCR. Relative quantification was performed using cel-miR-39-3p for normalization. **b** THP-1 cells were incubated with 20 μg/mL SHED-Exo or transfected with 50 nM of miRNA mimics as indicated. The miR-1246, miR-100-5p, and miR-92a-3p levels were determined by qRT-PCR. Relative quantification was performed using U6 small nuclear RNA for normalization. **c** Gene expression levels of autophagy markers Lc3b in THP-1 macrophages per group. **d** THP-1 cells were transfected with 50 nM of miR-1246 mimic and 100 nM of miR-1246 inhibitor. Western blot results and relative expression levels of LC3, AKT, ERK1/2, and STAT3 signaling pathway proteins per treatment group. *P < 0.05; **P < 0.01; ***P < 0.001; ****P < 0.0001

To gain further insight into SHED-derived exosome-mediated macrophage autophagy, we searched for significantly up-regulated miRNAs in SHED-derived exosomes using previously reported miRNA microarray analysis data and TargetScan database to identify miRNAs that could be potentially associated with AKT, ERK1/2, and STAT3 signaling pathways. Specifically, miR-1246, miR-100-5p, and miR-92a-3p were ranked in the top 3 of most enriched miRNAs in previously reported miRNA microarray analysis data of SHED-derived exosomes [38]. In addition, the TargetScan database analysis revealed that miR-1246, miR-100-5p, and miR-92a-3p could be associated with AKT, ERK1/2, and STAT3 signaling genes, including MTOR, WNT2B, DKK3, and WNT9B. To validate these, qRT-PCR analysis confirmed that miR-1246, miR-100-5p, and miR-92a-3p are enriched in SHED-derived exosomes (Fig. 8a). The results also demonstrated that miR-1246 were upregulated in THP-1 cells after treatment with SHED-Exos (Fig. 8b). Furthermore, transfection of miR-1246 in THP-1 macrophages specifically increased the gene and protein expression of LC3B (Fig. 8c, d), thereby indicating that SHED-derived exosomes containing miR-1246 promoting macrophage autophagy. Moreover, we found that both pretreatment of SHED-Exo and transfection of miR-1246 mimic significantly induced phosphorylation of AKT, ERK1/2, and STAT3 in macrophages, exosomes-induced phosphorylation expression was abrogated by miR-1246 inhibitor (Fig. 8d). These results further verify that SHED-Exo containing miR-1246 upregulated the expression of autophagy-related protein LC3 via the AKT, ERK1/2, and STAT3 signaling pathways, thereby regulating macrophage functions and promoting chronic wound healing with less of an itching response.

## Discussion

In this study, we investigated the cellular and molecular mechanisms underlying itching and cutaneous wound healing and showed that SHED-Exo induced autophagy in macrophages and contributed to the regulation of sensory neurons and wound closure. First, we found persistent itching and delayed cutaneous healing in an LPS-induced mouse model. Second, we showed that SHED-Exo have effects on wound closure and itching. Third, we found a close association between macrophage autophagy, wound healing, and itching. Finally, we demonstrated that SHED-Exo-enhanced autophagy regulates macrophage function by regulating the AKT, ERK1/2, and STAT3 signaling pathways. These findings indicated that SHED-Exo promoted wound healing with a lower itching sensation in an LPS-induced mouse model. This study provides the immunological and neurological underpinnings of high-quality wound healing capable of rapid wound closure and a lower sensation of itching.

Cutaneous wound healing is a coordinated physiological process involving the immune system, nervous system, and cells in skin tissue. It has been reported that itching may occur within a few days after wounding, which is a common and distressing problem affecting patients, resulting in sleep disorders and impaired psychosocial well-being [3, 4]. Therefore, both rapid wound closure and a comfortable healing process are the major goals for treating abnormal wounds. Inflammation is a key driver of itching and delayed wound healing; however, the underlying mechanism has not been studied [39, 40]. Abnormal wound healing has been reported to result in prolonged inflammation, impaired neovascularization, decreased collagen synthesis, and defective macrophage function. Furthermore, granulocytic function and chemotaxis defects lead to impaired healing, prone to infection [41]. The sensation of itching is a cascade associated with cross-communication between the immune and nervous systems. Neurons contribute to the recruitment of immune cells, and immune cells release inflammatory cytokines and activate receptors on sensory neurons [40]. Here, we discovered that direct wound administration of LPS led to delayed wound healing with itching. Consistent with these results, unlike excisional wounds in mice that resulted in significant regenerative healing with less itching, wounds in LPS-treated mice typically displayed delayed healing with heightened proinflammatory signals, reduced matrix deposition, and increased pruriceptive sensation. Therefore, the LPS-induced wound healing model could be useful for treating delayed healing and pruritis in abnormal wounds, such as wound infections, diabetic ulcers, and burns.

MSC-derived exosomes have the potential for therapeutic application in wound healing via tissue regeneration and regulation of immune response and inflammation [42]. It has been reported that secretion of MSCs with anti-inflammatory properties has beneficial effects on atopic dermatitis-related inflammatory lesions, such as pruritus [43], but the effects of exosomes on itching have not been studied. Previous studies have demonstrated that exosomes derived from MSCs could accelerate normal and diabetic wound healing [2, 44, 45]. However, only three clinical trials are currently in progress for cutaneous wound healing, according to ClinicalTrials.gov. This could be attributed to ethical issues and the high cost of the in vitro expansion of MSCs. Considering that SHED is isolated from a very accessible tissue resource from children and can provide large amounts of exosomes, we propose that SHED-derived exosomes could be a prominent candidate for clinical application. Here, we discovered the beneficial effects of SHED-Exo treatment on healing and pruritus in an LPS-induced wound model. In the present study, we not only showed that SHED-Exo improved LPS-induced wound healing, but also showed for the first time that SHED-Exo suppress the sensation of itching during abnormal wound healing. Interestingly, treatment with 3-MA significantly reversed the effects of SHED-Exo on wound closure and pruritus, suggesting that the effect of SHED-Exo was associated with autophagy. We also found that RAPA mimicked the effects of exosomes, but not dramatically. Therefore, we speculated that autophagy is a likely approach to wound closure and pruritus when the skin is recovering from injury.

The type of cells responsible for autophagy during wound healing and whether it influences inflammation at the lesion site were determined. We found that macrophages are the main cell type in the skin during the process of wound healing. Macrophages have been shown to play a key role in the regulation of all stages of tissue repair. Distinct macrophage phenotypes exhibit complex roles in triggering inflammatory responses while limiting tissue damage [6]. Macrophages are a crucial source of inflammatory mediators that recruit leukocytes and stimulate phagocytosis during the early inflammatory phase. Thereafter, macrophages respond to anti-inflammatory mediators, such as IL-10 converted from a proinflammatory to reparative phenotype, activating local progenitor cells and stem cells that participate in tissue repair. Considering these results, our results showed that increased macrophage autophagy significantly promoted the release of the anti-inflammatory mediator IL-10 instead of the proinflammatory mediator TNF-α in skin tissue. Impaired macrophage autophagy can lead to inflammasome-mediated proinflammatory cytokine production and deficiencies in the generation of inhibitory macrophages [46]. In this study, we expanded the list of macrophage autophagy to wound closure and itching by demonstrating that macrophages regulate parenchymal cell proliferation and sensory neuron sensitivity. It has been reported previously that macrophage-specific autophagy regulates mitochondrial dysfunction to control inflammation via the MAPK-NF-κB signaling pathway [47]. The regulation of autophagy by resident macrophages cleared neuron-released α-synuclein to protect neuron functions [48, 49]. Moreover, autophagy in peripheral macrophages mediates the control of polarization through the NF-κB and mTOR signaling pathways [50]. Therefore, we speculated that macrophage-specific autophagy could regulate the proliferation of keratinocytes and fibroblasts and neuronal sensitivity by controlling cutaneous macrophage polarization and function. Our data showed an interaction between cutaneous macrophages, parenchymal cells, and peripheral sensory neurons, suggesting the importance of cross-communication between the immune and nervous systems in the skin.

Emerging evidence has shown that MSC-derived exosomes exert a critical immunomodulatory function in tissue regeneration [51]. Our current study demonstrated that the promotion of wound closure and inhibition of itching by SHED-Exo in an LPS-induced wound model could be explained by the anti-inflammatory effects of exosomes, including exosome-mediated decreases in inflammation and M2 macrophage polarization. Recent studies have addressed the cross-talk between MSCs and macrophages, by which macrophage autophagy and phenotype are regulated [52, 53]. However, we are the first to demonstrate that SHED-Exo enhance autophagy and protect the function of mitochondria, which dampens neuron sensitivity and facilitates the decrease in tissue inflammation. Reducing autophagy in monocytes by reducing ATG5 activated the SQSTM1-KEAP1-NFE2L2 axis, leading to an increase in the antioxidant response, which further affected mitochondrial function and biogenesis [54]. Macrophage autophagy limits the release of inflammatory cytokines, such as IL-1β, by restraining NLRP3-inflammasome activation via the NF-κB-p62-mitophagy pathway [55]. Autophagy also manipulates macrophage polarization via the ROS/ERK signaling pathway and NLRP3 function [56, 57]. Therefore, these findings support the supposition that SHED-derived exosome-manipulated macrophage autophagy that controls the macrophage phenotype could provide clinical strategies for better wound healing with less sensation of pruritus.

We further confirmed that SHED-Exo increased the LC3 II/LC3 I ratio by activating the AKT/FoxO1, ERK1/2, and STAT3 signaling pathways, which also play crucial roles in macrophage activation and polarization. The AKT/FoxO1 pathway has been shown to regulate autophagy and polarization [58–60]. It has been reported that FoxO1 is translocated from the nucleus to the cytosol when p-FoxO1 in LPS-stimulated macrophages was increased, and macrophage polarization towards the M2-like phenotype was observed [60]. Similarly, we demonstrated that LY294002 blocked increased FoxO1 phosphorylation induced by SHED-Exo. Furthermore, ERK1/2, an upstream negative regulator of mTOR/p70S6K, stimulates mTOR activation, thus promoting autophagy [61, 62]. ERK1/2 inhibitor U0126 decreased ERK1/2 phosphorylation and attenuated autophagy flux in LPS-induced macrophages. Recent studies revealed that Stat3 positively regulates autophagy via mitochondrial translocation and that Stat3-mediated autophagy activation can be blocked by 3-MA [61]. STAT3 activation releases FoxO1 and FoxO3 from cytoplasmic anchorage and mediates the proautophagic signaling pathway through p-FoxO1 and p-FoxO3 through translocation to the nucleus [63].

MSC-derived exosomes contained abundant miRNAs that function largely via horizontal transfer, thereby leading to phenotypic modification and reprogramming of target cells [52]. In our current study, not only SHED-Exo treatment, but also transfection of miR-1246 mimic in THP-1 cells significantly induced phosphorylation of AKT, ERK1/2, and STAT3 significantly promoted LC3 I and II expression, whereas blocking of SHED-Exo effects by miR-1246 inhibitor decreased LC3 expression (Fig. 8d). Database analysis revealed that miR-1246 targeting candidate gene MTOR was an autophagy associated gene [64], suggesting that miR-1246 might directly target MTOR to control autophagy regulation. Our present findings demonstrate that SHED-Exo containing miR-1246 induced autophagy activation is mediated through the AKT/FoxO1, ERK1/2, and STAT3 pathways.

It should be noted that the AKT/FoxO1, ERK1/2, and STAT3 pathways may not be the sole regulator of autophagy in SHED-Exo-regulated macrophages. Furthermore, many cargos loaded in SHED-Exo regulate phenotypic modification and reprogramming of macrophages. The principle underlying the structural and functional imbalances of macrophages during inflammatory injury has not been fully studied. It is possible to find a new way to solve the problem of chronic wound healing in these areas.

Our study provides evidence that SHED-Exo, which regulates macrophage function, is beneficial for high-quality wound healing. SHED is an ideal source of exosomes for clinical applications because of its easy accessibility, excellent pluripotency, and prominent exosome production ability. Collectively, SHED-derived exosomes may be a promising therapeutic tool against impaired wound healing and other inflammatory disorders.

## Materials and methods

### Cell isolation and culture

SHED were isolated and collected from non-caries-exfoliated human deciduous teeth (4–10-year old; six males and six females, without oral or systematic diseases) after obtaining informed consent, and the experimental protocol was approved by the Ethics Committee of Hospital of Stomatology, Sun Yat-sen University. Briefly, the pulps from deciduous teeth were minced and digested with 3 mg/mL collagenase type I and 4 mg/mL dispase (Gibco-BRL) and cultured in DMEM (Gibco-BRL) containing 10% FBS, 100 units/mL streptomycin (HyClone), and 100 units/mL penicillin (HyClone). THP-1 cells were purchased from Cyagen Biosciences and cultured in RPMI 1640 (Gibco-BRL) containing 10% FBS, 100 units/mL streptomycin, and 100 units/mL penicillin. THP-1 cells were differentiated into macrophage-like cells with 100 ng/mL phorbol 12-myristate 13-acetate (PMA, Sigma-Aldrich) and incubated for 48 h. The human epidermal keratinocyte cell line HaCaT was obtained from the Chinese Academy of Sciences and cultured in DMEM containing 10% FBS, 100 units/mL streptomycin, and 100 units/mL penicillin. The human foreskin fibroblast cell line HFF was obtained from Procell Life Science & Technology and maintained in DMEM supplemented with 10% FBS, 100 units/mL streptomycin, and 100 units/mL penicillin. The neuroblastoma cell line SH-SY5Y was purchased from Procell Life Science & Technology and maintained in DMEM/F12 (Thermo Fisher Scientific) medium supplemented with 15% FBS, 100 units/mL streptomycin, and 100 units/mL penicillin.

### Characterization of SHED

For the colony-forming unit assay, 1000 cells were seeded into 10 cm dishes. The culture medium was changed once every 3 d. After 14 d, cells were fixed in 4% paraformaldehyde, followed by staining with 0.1% crystal violet solution (Sigma-Aldrich). For the osteogenic induction assay, when the cell density reached 80%, the medium was replaced with osteogenic induction medium (DMEM containing 10% FBS, 10 mM β-glycerophosphate, 10 nM dexamethasone, and 50 μg/mL ascorbic acid), which was changed every 3 d. After 2 weeks of induction, the cells were fixed in 4% paraformaldehyde and stained with 1% alizarin red. For the lipogenesis induction assay, when the cell reached 80% confluence, the medium was replaced with lipogenesis induction solution A (DMEM complete medium with 10% FBS, 100 U/mL penicillin, 100 μg/mL streptomycin, 1 μM dexamethasone, 0.2 mM indomethacin, 10 μg/mL insulin, 0.5 mM IBMX), which was incubated for 3 d, then replaced with lipogenesis induction solution B (DMEM complete medium with 10% FBS, 100 U/mL penicillin, 100 μg/mL streptomycin, and 10 μg/mL insulin) and maintained for 1 d. After 3 weeks of induction, the cells were fixed in 4% paraformaldehyde and stained with Oil Red O. The specific surface antigens of SHED in cultures were characterized using flow cytometry. Cells were stained with phycoerythrin (PE)-conjugated antibodies against CD34, CD44, CD45, CD90, CD105, and HLA-DR (BD Biosciences) for 30 min in the dark. Thereafter, the cells were analyzed using a flow cytometer (Beckman). For tissue origin identification, when SHED reached 60% confluence, the cells were fixed with 4% paraformaldehyde, permeabilized with 0.1% Triton X-100, and blocked with 5% BSA for 1 h. Cells were incubated with mouse anti-human vimentin antibody (1:200, Santa Cruz) or mouse anti-human keratin antibody cytokeratin 18 (1:500, Abcam) in a wet box overnight at 4 °C. After incubation with fluorescently labeled secondary antibody (1:500 dilution for 1 h) followed by Hoechst 33258 (Life Technologies), cell fluorescence was observed under a fluorescence microscope (Carl Zeiss).

### Isolation and characterization of exosomes

Exosomes were prepared according to the International Society of Extracellular Vesicles (ISEV) recommendations [26]. For exosome purification from cell culture supernatants, SHED were cultured in media supplemented with 10% exosome-depleted FBS. FBS-derived exosomes were predepleted by overnight centrifugation at 100,000×*g*. SHED-derived exosomes were isolated from conditioned media using ultracentrifugation method as previously described [65]. Briefly, culture supernatants were centrifuged serially at 300×*g* for 10 min, 2000×*g* for 20 min and 10,000×*g* for 30 min 4 °C to remove cells, dead cells and cell debris, respectively. The supernatants were centrifuged at 100,000×*g* for 70 min (Beckman Coulter) to collect the exosome pellet. The Nanoparticle Characterization System (NanoSight, Malvern Instruments) and ZetaView PMX 120 Zeta Potential Analyzer (Particle Metrix, Meerbusch, Germany) was used to measure the size distribution and zeta potential of exosomes. The morphological characteristics of exosomes were detected using a Tecnai G2 Spirit Twin transmission electron microscope (FEI Company). The expression of surface markers, including CD63 (Abcam), CD81 (Santa Cruz), CD9 (Abcam), TSG101 (Abcam), ALIX (Abcam), α-tubulin (Cell Signaling Technology), Albumin (Abcam), on exosomes was analyzed by western blotting. SHED-Exo were incubated with FITC-conjugated anti-CD63 (BD Biosciences), FITC-conjugated anti-CD81 (BD Biosciences), and FITC-conjugated anti-CD9 (BD Biosciences) at 37 °C for 30 min in the dark, and then washed twice with 1 mL PBS. The pellet was resuspended in 50 μL PBS for nano-flow cytometer analysis (NanoFCM).

### Wound healing model in mice

The animal experiments were approved by the Animal Ethical and Welfare Committee of Sun Yat-Sen University (No. SYSU-IACUC-2021-000429). Mice were randomly divided into the following groups (n = 5): the (1) control group, (2) LPS group, (3) LPS and SHED-derived exosomes group (LPS + Exo); (4) LPS, SHED-derived exosomes, and 3-MA group (LPS + Exo + 3MA), and (5) LPS + rapamycin group (LPS + RAPA). An excisional full-thickness skin wound splinting model was developed. Full-thickness round excision wounds (1 cm in diameter) were created on the back of the mice. The cutaneous wounds were subcutaneously injected with 0.5 mg/kg LPS, 10 mg/kg autophagy inhibitor 3MA, 1 mg/kg autophagy promoter rapamycin, or 10 mg/kg SHED-derived exosomes dissolved in 200 μL PBS. A series of digital photographs of the cutaneous wounds was taken during the wound healing process, and a standard ruler was used as a scale. At the indicated time points, wound size was calculated on photographs using Adobe Photoshop Elements 14 software. Changes in the wound area are expressed as a percentage of the initial wound area.

### Histological and immunofluorescence analysis

After 5 and 10 d of healing, the wound skin of the mice was cut off and fixed in 4% buffered paraformaldehyde. Then, the samples were embedded in paraffin after dehydration with ethanol, and sections (5 μm thick) were stained with hematoxylin and eosin (H&E) and Masson’s trichrome for histological analysis. Immunohistochemical staining for TRPV4 (1:100, Abcam) and CD31 (1:500, Proteintech) were performed as described previously [66]. For immunofluorescence staining, paraffin-embedded tissue sections were dewaxed, antigen repair was performed, the tissue slides were permeabilized with 0.1% Triton X-100, and the tissue sections were blocked in 5% BSA at room temperature for 2 h. The specific primary antibodies for mouse LC3B (1:500, Abcam) and CD68 (1:1000, Abcam) were added to the sections in a wet box overnight at 4 °C. The sections were then incubated for 2 h with fluorescently labeled secondary antibodies (Alexa Fluor 488 and Alexa Fluor 594, Abcam), followed by incubation with 0.5 μg/mL Hoechst 33258 (Life Technologies) for 5 min.

### Pruriceptive behavior measurement

For the measurement of pruriceptive behaviors, mice were placed in clear plastic cages, allowing free movement. Behavioral itch-related responses were videotaped during the experiment. Behavioral experiments and data analysis were performed in a blinded fashion. The number of scratch bouts around the wound was counted for 30 min.

### Total RNA extraction and reverse transcription-quantitative PCR (RT-qPCR)

Mouse skin samples and THP-1 cells were homogenized using TRIzol reagent (Ultrapure RNA Kit, CW Biotech). Total RNA was isolated using an RNA isolation kit (Ultrapure RNA Kit, CW Biotech) and transcribed into cDNA using a Reverse Transcriptase M-MLV kit (TaKaRa). RT-qPCR was conducted using the SYBR Green PCR Master Mix kit (Roche) with the following conditions: 95 °C for 10 min, 40 cycles of denaturation at 95 °C for 15 s, annealing at 60 °C for 20 s, and extension at 72 °C for 20 s. The primer sequences used in this study are listed in Table 1. The 2^−ΔΔCt^ method was used to calculate the expression level of the target gene, and GAPDH was used as an internal control.

**Table 1:** Primer sequences used in reverse transcription quantitative PCR (RT-qPCR)

Gene	Species	Sequence (5′–3′)	
Lc3	Mouse	Forward primer	CGTCCTGGACAAGACCAAGT
		Reverse primer	ATTGCTGTCCCGAATGTCTC
Beclin	Mouse	Forward primer	AATCTAAGGAGTTGCCGTTATAC
		Reverse primer	CCAGTGTCTTCAATCTTGCC
P62	Mouse	Forward primer	GCTGCCCTATACCCACATCT
		Reverse primer	CGCCTTCATCCGAGAAAC
Nos2	Mouse	Forward primer	AATCTTGGAGCGAGTTGTGG
		Reverse primer	CAGGAAGTAGGTGAGGGCTTG
Tnf-α	Mouse	Forward primer	TCGAGTGACAAGCCTGTAGCC
		Reverse primer	TTGAGATCCATGCCGTTGG
Il-10	Mouse	Forward primer	GCCAGAGCCACATGCTCCTA
		Reverse primer	GTCCAGCTGGTCCTTTGTTTG
GAPDH	Mouse	Forward primer	TGGCCTTCCGTGTTCCTAC
		Reverse primer	GAGTTGCTGTTGAAGTCGCA
CD68	Human	Forward primer	GGAAATGCCACGGTTCATCCA
		Reverse primer	TGGGGTTCAGTACAGAGATGC
Il31ra	Human	Forward primer	AACATAGCGAAAACTGAACCACC
		Reverse primer	GCCAACTCAGGCTTTATCCATTC
Nppb	Human	Forward primer	TGGAAACGTCCGGGTTACAG
		Reverse primer	CTGATCCGGTCCATCTTCCT
Trpv4	Human	Forward primer	CTACGGCACCTATCGTCACC
		Reverse primer	TTAGGCGTTTCTTGTGGGTCA
Trpv1	Human	Forward primer	CTGCCCGACCATCACAGTC
		Reverse primer	CTGCGATCATAGAGCCTGAGG
Lc3b	Human	Forward primer	GATGTCCGACTTATTCGAGAGC
		Reverse primer	TTGAGCTGTAAGCGCCTTCTA
GAPDH	Human	Forward primer	GGAGCGAGATCCCTCCAAAAT
		Reverse primer	GGCTGTTGTCATACTTCTCATGG

### Western blot analysis

Mouse skin samples and THP-1 cells were washed with PBS and lysed in RIPA buffer (50 mM Tris–HCl pH 7.4, 150 mM NaCl, 1%TritonX-100, 0.5% sodium deoxycholate, 0.1% SDS, and protease inhibitor cocktail) on ice for 30 min. Protein samples were separated by 10% sodium dodecyl sulfate-polyacrylamide gel electrophoresis (SDS-PAGE) and then transferred onto nitrocellulose (NC) membranes. The membranes were incubated with the appropriate primary antibodies: anti-LC3B (1:1000, Cell Signaling Technology), anti-Beclin1 (1:1000, Cell Signaling Technology), anti-P62 (1:1000, Cell Signaling Technology), anti-pan-Akt (1:1000, Cell Signaling Technology), anti-Phospho-Akt (1:1000, Cell Signaling Technology), anti-FoxO1 (1:1000, Cell Signaling Technology), anti-Phospho-FoxO1 (1:1000, Cell Signaling Technology), anti-Phospho-ERK1/2 (1:2000 Cell Signaling Technology), anti-Erk1/2 (1:1000, Cell Signaling Technology), anti-Phospho-STAT3 (1:2000, Cell Signaling Technology), anti-STAT3 (1:2000, ABclonal), and anti-α-tubulin (1:2000, Cell Signaling Technology). After incubation with secondary antibodies for 1 h at room temperature, the blotted membranes were visualized using an Odyssey two-color infrared laser imaging system (LI-COR Biosciences) and quantified using ImageJ software and normalized to α-tubulin.

### Cell proliferation detection

To measure cell proliferation, cells were seeded in 96-well plates at a density of 10^4^ cells/well. At different time points (1, 3, or 5 d), the cells in each well were incubated with 100 μL DMEM and 20 μL 3-(4,5-dimethylthiazol-2-yl)-5-(3-carboxymethoxyphenyl)-2-(4-sulfophenyl)-2H-tetrazolium (MTS) (Promega) solution for 3 h at 37 °C. The optical density was measured at 490 nm.

### Cell wound scratch assay

Cells were seeded in 6-well plates for migration testing. When the cells reached 90% confluence, the monolayer of cells was scratched using a sterile 200 μL tip, and then the cells were cultured under standard conditions for 48 h. Migration of cells was captured at 0, 24, and 48 h using a phase-contrast microscope.

### Flow cytometry

Approximately 1 × 10^6^ THP-1 cells were seeded in 6-well plates. After PMA stimulation, cells were incubated with PE-conjugated anti-CD86 (1:20, BioLegend) and APC-conjugated anti-CD206 (1:20, BioLegend) at 37 °C for 20 min in the dark. The fluorescence intensity was measured using a flow cytometer (Beckman).

### Measurement of oxidative stress

THP-1 cells were seeded in a 6-well plate at a density of 1 × 10^6^ cells/well and then induced in RPMI 1640 medium with 100 ng/mL PMA for 48 h at 37 °C and 5% CO_2_. Cellular ROS was measured using a DCFH-DA probe (Sigma-Aldrich), as described previously [67]. The fluorescence intensity was measured using a flow cytometer (Beckman).

### Mitochondrial ultrastructure and mitochondrial membrane potential assay

Ultrastructural analysis of mitochondria was performed using transmission electron microscopy (TEM). Cell pellets were fixed in 2.5% glutaraldehyde in 0.1 M phosphate buffer for 2 h. Samples were placed on 100 mesh copper grids, post-stained with 1% uranyl acetate and 0.4% lead citrate, and examined at 80 kV using a Tecnai G2 Spirit Twin Transmission Electron Microscope (FEI Company). Mitochondrial membrane potential was measured using a JC-1 fluorescent probe assay kit (Beyotime Biotech). JC-1 work reagents were prepared according to the manufacturer’s protocols. Cells were incubated for 20 min at 37 °C in the dark after adding reagents to the cell medium. Cell fluorescence was observed using an LSM 780 confocal microscope (Carl Zeiss).

### Cell transfection

THP-1 cells were transfected with 50 nM miRNA mimics, 100 nM miRNA inhibitors, or the corresponding control oligonucleotide NC mimic using a RiboFECT CP Transfection Kit (RiboBio) according to the manufacturer’s instructions. Following incubation for 48 h, qRT-PCR and western blot were performed to validate of mRNA and protein change.

### Statistical analysis

SPSS 20.0 software package was used for statistical analysis. All data are presented as mean values ± SD. For the comparison of three or more groups, a one-way analysis of variance was used, and post hoc Bonferroni tests were performed for multiple comparisons. Statistical significance was set at p < 0.05.

## Supplementary Information

**Additional file 1: Figure S1.** Characterization of SHED. **Figure S2.** Characterization of SHED-Exo. **Figure S3.** Characterization of macrophages. **Figure S4.** SHED-derived exosomes uptake efficiency in macrophages at the wound site.

## Data Availability

The data supporting the findings of this study are available from the corresponding author upon reasonable request.
