# Supplementary material for: SHED-derived exosomes promote LPS-induced wound healing with less itching by stimulating macrophage autophagy
Source: J Nanobiotechnology. 2022 May 21;20:239. doi: 10.1186/s12951-022-01446-1 (PMC9124392; doi:10.1186/s12951-022-01446-1)
Supplement: Supplementary file 1 — Additional file 1: Figure S1. Characterization of SHED. Figure S2. Characterization of SHED-Exo. Figure S3. Characterization of macrophages. Figure S4. SHED-derived exosomes uptake efficiency in macrophages at the wound site. [file 12951_2022_1446_MOESM1_ESM.docx]

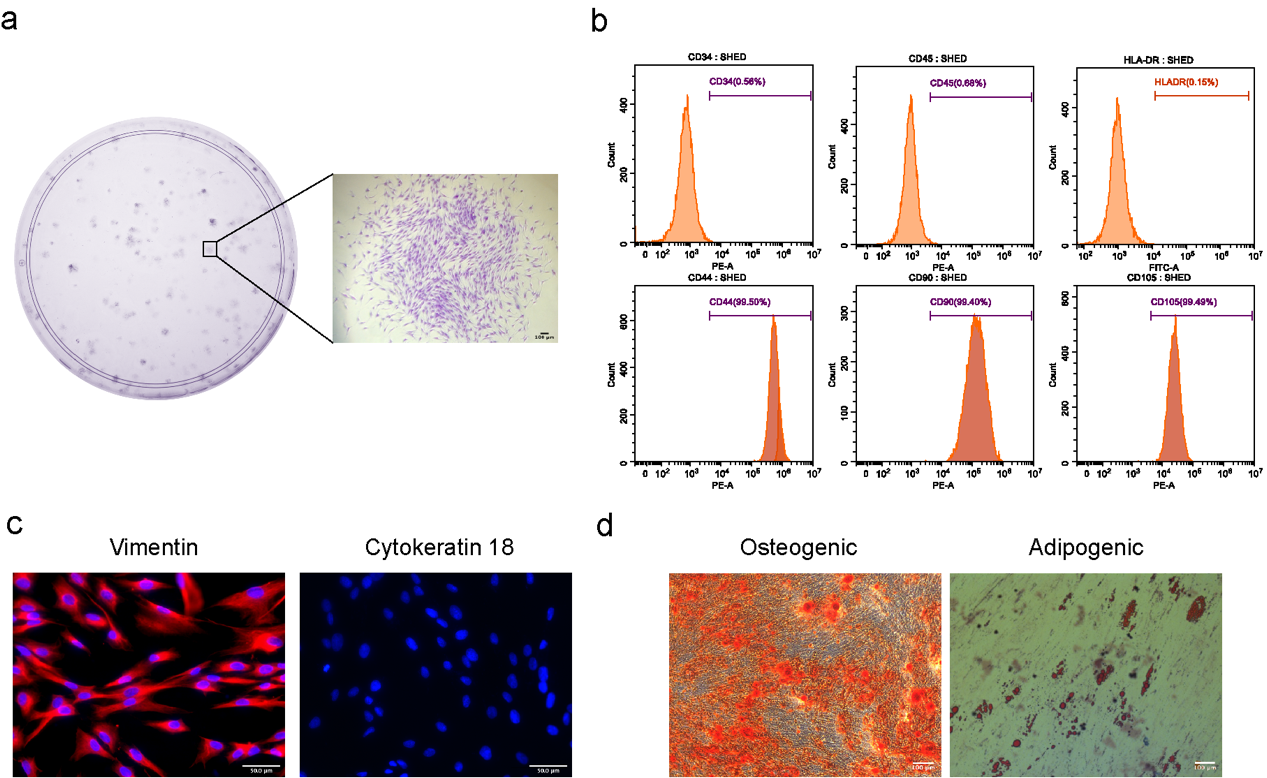


Fig.S1. Characterization of SHED.

(a) Colony formation assay. Scale bars, 100 µm. (b) Surface markers identification. (c) Tissue origin identification. Scale bars, 500 µm. (d) Multiple differentiation ability demonstrated by alizarin red (left) or Oil Red O (right) staining. Scale bars, 100 µm.


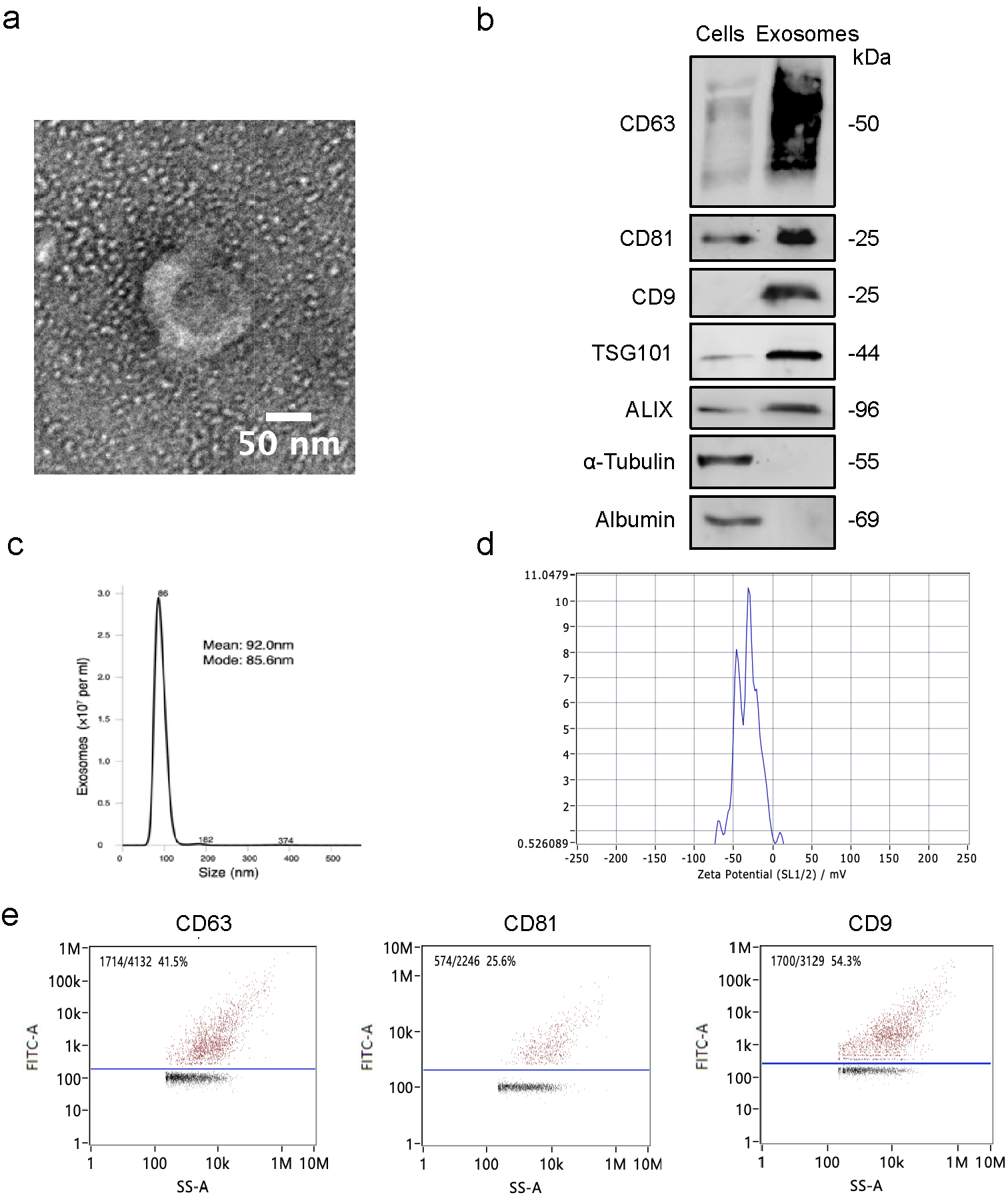


Fig. S2. Characterization of SHED-Exo.

(a) The morphology of SHED-Exo was analyzed by TEM. Scale bars, 50 nm. (b) The surface markers of exosomes were evaluated by western blotting. SHED cells were used as a control. (c) The diameters of SHED-Exo were measured by NTA analysis. (d) Zeta potential measurements of SHED-Exo were detected by ZetaView. (e) The expression of CD63, CD81, and CD9 of SHED-Exo were evaluated by nano-flow cytometry.


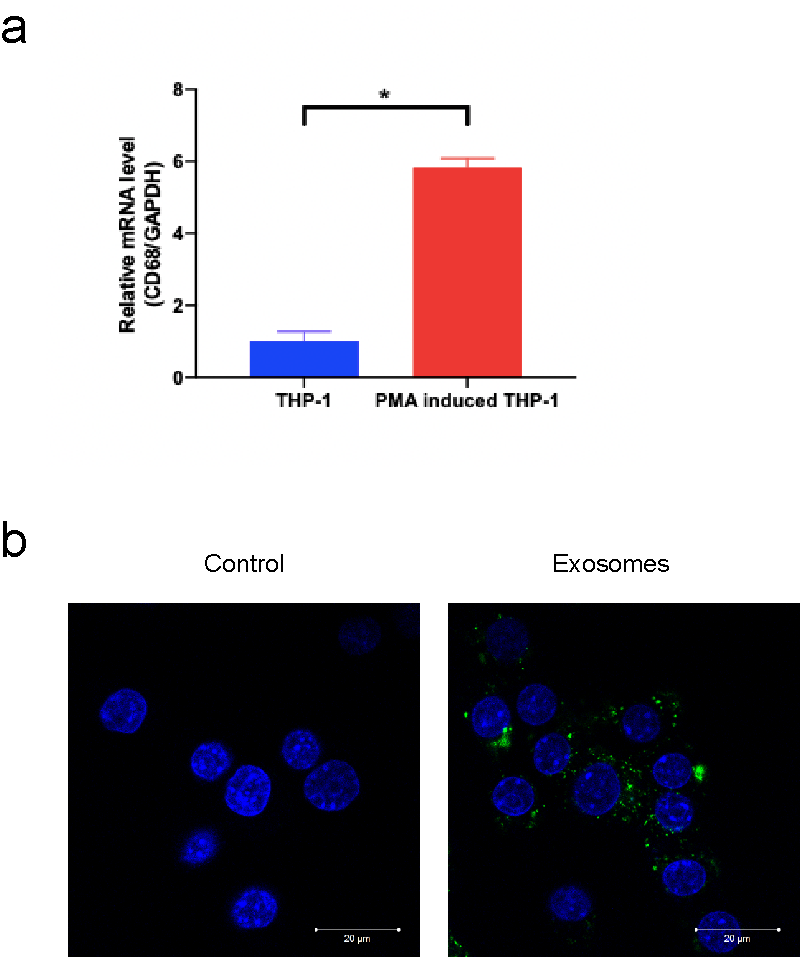


Fig. S3. Characterization of macrophages.

(a) RT-qPCR analysis of the expression of the macrophage marker CD68. **P* < 0.05; ***P* < 0.01; ****P* < 0.001. (b) Representative immunofluorescence images showing the internalization of PKH67-labeled SHED-Exo (green) by THP-1 macrophages. Scale bars, 20 µm.


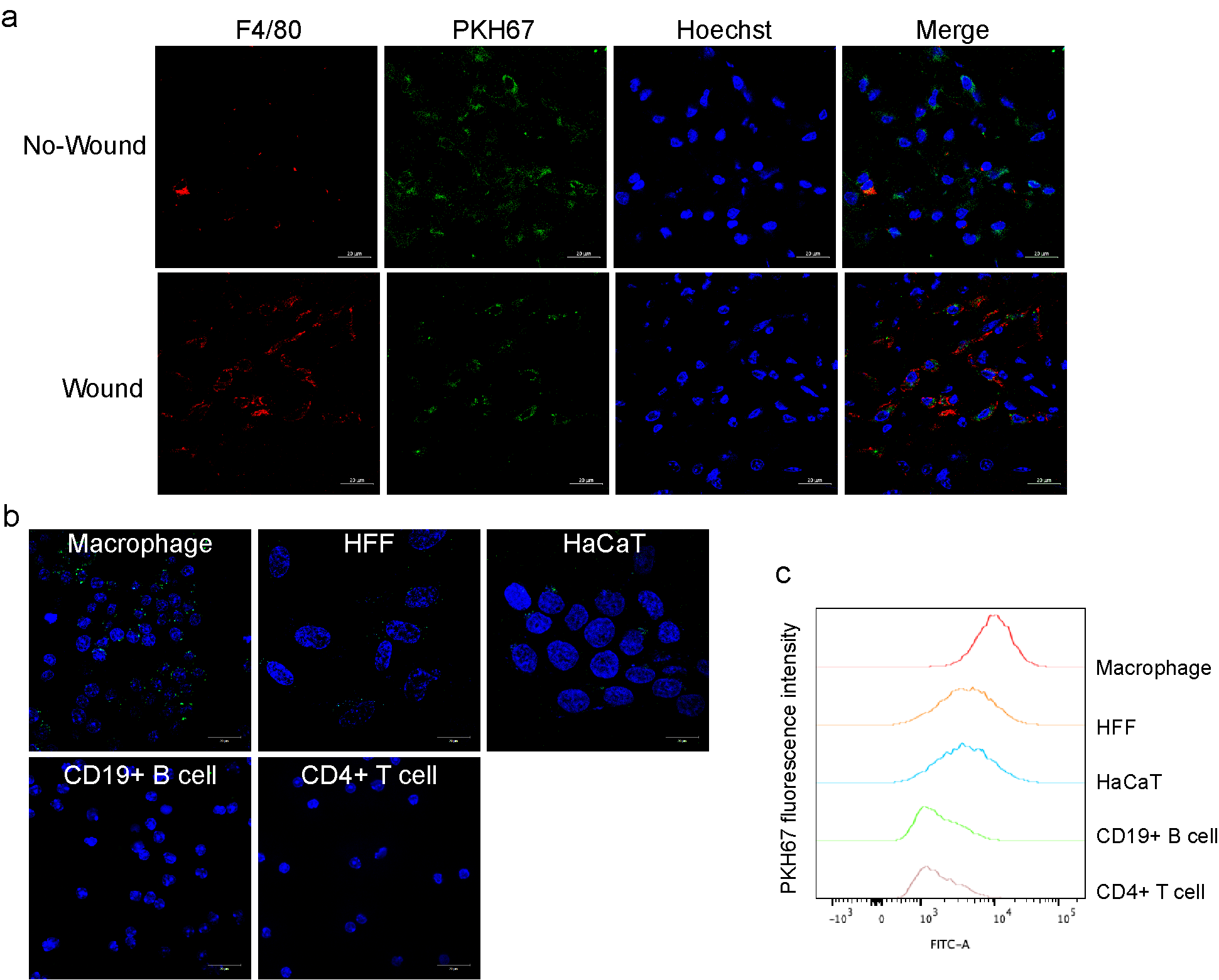


Fig. S4. SHED-derived exosomes uptake efficiency in macrophages at the wound site.

(a) The cutaneous wounds were subcutaneously injected with 10 mg/kg PKH67-labeled SHED-Exo (green). Wound tissue sections on 48 h post-operation were stained with F4/80 as a marker for macrophages. Representative images of skin tissue sections stained for immunofluorescent detection of F4/80 (red), and hoechst (blue). Scale bars, 20 µm. (b) Confocal images of various types of cells after 4 h incubation with 20 μg/ml of PKH67-labeled SHED-Exo. Images of PKH67-labeled SHED-Exo (green) with Hoechst (blue) were visualized by merging the confocal images. Scale bars, 20 µm. (c) Fluorescence intensity of PKH67-labeled SHED-Exo in various types of cells was detected by flow cytometry.
